# Noncommunicating cystic pancreatic lesions (i.e. serous and mucinous cystadenoma + rare lesions)

**DOI:** 10.1186/1470-7330-14-S1-O27

**Published:** 2014-10-09

**Authors:** Jay P Heiken

**Affiliations:** 1 Mallinckrodt Institute of Radiology, Washington University School of Medicine, St. Louis, Missouri 63110, USA

## 

*Mucinous cystic neoplasm (MCN)* occurs predominantly in women (>95%) with a mean age of approximately 45 years and most commonly is located in the pancreatic body or tail. The mass is lined by mucin-producing columnar epithelium with a fibrous ovarian-type stroma. The epithelial lining can contain various degrees of atypia, ranging from adenoma to invasive carcinoma. The CT and MR appearance is that of a thick walled cystic mass with septa and/or mural nodules. The contour of the mass is smooth, and the wall or septa may contain calcification. Endoscopic ultrasound with fine needle aspiration of the cyst fluid to assess CEA level may be helpful in distinguishing MCN from serous cystadenoma. CEA <5 ng/mL favors serous cystadenoma, whereas CEA >192 ng/mL favors MCN, with an accuracy of approximately 80% [1]. The overall risk of malignancy in one series was 17.5%, and all malignant tumors were either >4 cm or contained mural nodules [2]. Treatment of MCN is surgical resection, and patients without extracapsular or diffuse intracapsular infiltration have an excellent prognosis [2].

*Serous cystadenoma (SCA)* also occurs predominantly in women (approximately 75%), but the mean age is older (mid 50s to early 60s). It is lined by glycogen-rich cuboidal epithelium and is considered a benign neoplasm, although rare cases of malignant SCA have been reported. The typical appearance is that of a lobulated mass consisting of numerous tiny cysts which give it a honeycomb appearance. Larger lesions may contain a central stellate scar and calcifications. Oligocystic or macrocystic SCA is an uncommon variant comprised of a small number of larger cysts. Distinction between macrocystic SCA and MCN can be made based on the lobulated contour of SCA, its multiple clustered cyst configuration and homogeneity of each locule on T1 weighted MR images [3]. Surgical resection generally is reserved for symptomatic patients, although some authors recommend periodic imaging to assess the rate of growth, with consideration given to surgical resection of lesions with doubling time of <6 years [4].

*Solid pseudopapillary neoplasm (SPN)* is a rare low-grade malignancy that predominantly affects younger women (>80%), with a median age 30-38 years [5,6]. It is not a true cystic neoplasm in that the cystic component lacks an epithelial lining. Rather, the cystic component represents necrotic degeneration of the mass, with various amounts of internal blood and debris. SPN appears as a well-circumscribed round or oval thick-walled mass with solid and cystic components, but it can appear completely solid or completely cystic. CT demonstrates calcification in nearly half of SPNs [6]. MR often demonstrates a thick T2 hypointense or enhancing rim and internal blood products. Treatment is surgical resection, and the prognosis is excellent, with metastases developing in <5% of patients [6].

*Cystic neuroendocrine neoplasm* of the pancreas most commonly occurs in patients with multiple endocrine neoplasia type I and usually is nonfunctional [7]. It generally appears as a cystic mass with an enhancing rim and may contain septa or a solid component. Treatment usually is surgical resection, although observation may be a viable alternative approach [8].
